# The Effects of Tai Chi Exercise for Patients with Type 2 Diabetes Mellitus: An Overview of Systematic Reviews and Meta-Analyses

**DOI:** 10.1155/2022/6587221

**Published:** 2022-06-28

**Authors:** Hongshuo Shi, Shaoting Wang, Yufeng Zhang, Pulin Liu, Chengda Dong, Dan Wang, Guomin Si, Wenbo Wang, Yujie Li

**Affiliations:** ^1^Shandong University of Traditional Chinese Medicine, Jinan, China; ^2^Dongying People's Hospital, Dongying, China; ^3^Provincial Hospital Affiliated to Shandong First Medical University, Jinan, China; ^4^The Second Affiliated Hospital of Shandong University of Traditional Chinese Medicine, Jinan, China

## Abstract

**Objectives:**

Tai chi (TC) is a potential complementary treatment for type 2 diabetes mellitus (T2DM). This overview systematically summarizes and evaluates the existing evidence of TC in the treatment of T2DM.

**Methods:**

Systematic reviews (SRs)/meta-analyses (MAs) on TC interventions for T2DM were comprehensively searched in seven databases. Methodological quality, risk of bias, reporting quality, and quality of evidence were assessed using the Assessment of Multiple Systematic Reviews 2 (AMSTAR-2), the Risk of Bias in Systematic (ROBIS) scale, the list of Preferred Reporting Items for Systematic Reviews and Meta-Analysis (PRISMA), and the Grading of Recommendations Assessment, Development, and Evaluation (GRADE) system.

**Results:**

Eight published SRs/MAs were included in our study. Based on the methodology and quality of evidence assessment, all SRs/MAs are considered to be of very low quality, and only 1 SR/MA has been assessed as low risk of bias, and none of the SR/MA has been fully reported on the checklist. A total of 65 outcome indicators extracted from the included SRs/MAs were evaluated, and only 1 item was assessed as high quality.

**Conclusions:**

TC may be an effective and safe complementary treatment for T2DM. However, this conclusion must be treated with caution because the quality of the evidence provided by the included SRs/MAs is generally low.

## 1. Introduction

As a chronic metabolic disease, type 2 diabetes mellitus (T2DM) is characterized by elevated blood glucose levels, which induces disturbances in glucose, fat, and protein metabolism in the body [1]. The pathophysiological hallmark of T2DM is insulin resistance, accompanied by decreased insulin secretion due to pancreatic *β* cell dysfunction, and oxidative stress (OS) is also considered to be a major hallmark of the pathogenesis and progression of T2DM [2]. T2DM accounts for 90% of all diabetes cases, and the International Diabetes Federation (IDF) estimates that the number of people with type 2 diabetes worldwide is expected to reach 578 million by 2030 and 700 million by 2045 [3]. T2DM is generally associated with a higher risk of vascular complications, including macrovascular complications (e.g., cardiovascular complications) and microvascular complications (including neuropathy, nephropathy, and retinopathy) [4]. The current mainstream treatments for T2DM include oral hypoglycemic drugs and subcutaneous insulin injections. In addition, body movement constitutes a significant part of a diabetes management program and plays a specific role in preventing diabetes complications and managing blood sugar in patients with T2DM [5].

OS occurs when the accumulation of the by-products of oxygen metabolism eventually exceeds the antioxidant capacity [6]. Experimental and clinical studies have shown that OS is involved in the pathogenesis of cardiovascular disease, carcinogenesis, and other diseases. It is known to play a key role in the etiology and pathophysiology of diabetes [7]. Mitochondrial dysfunction and dysregulation of prooxidase appear to be major factors involved in chronic reactive oxygen species (ROS) generation, resulting in a chronic OS state [2]. Chronic hyperglycemia is considered to be a major contributor to the development of microvascular and macrovascular complications in type 2 diabetes and is known to cause DNA, lipid, and protein damage, the extent of which is related to the hyperglycemia-induced reactive oxygen species production and the degree of OS [8]. Physical exercise intervention induces an adaptive response characterized by a reduction in markers of OS damage and an increase in the body's antioxidant response [9].

Originated in ancient China, TC has a history of thousands of years. It was formed under the guidance of traditional Chinese medicine theories and Chinese folk martial arts, and it is a gem of the Chinese nation [10]. The pathogenesis of T2DM is closely related to the physical and mental state of the individual. TC is a movement that emphasizes the unity of energy, breath, and spirit and combines physical activity with breathing [11]. A meta-analysis showed that regular TC exercise increased superoxide dismutase and catalase levels while decreasing lipid peroxide levels [12]. Therefore, TC plays an active role in glycemic control and OS regulation in T2DM patients, and the ADA recommends TC as a mind-body therapy for T2DM patients to increase balance, muscle strength, and flexibility [13].

Systematic review (SR)/meta-analysis (MA) is an important tool for evidence-based clinical work, but its methods must strictly follow a series of guidelines to minimize the possibility of deviation in answering specific questions [14]. A growing number of SRs/MAs based on TC interventions for T2DM have shown that TC can reduce fasting glucose (FGB) and glycated hemoglobin (HbA1c) and improve the quality of life in patients with T2DM. However, the methodological and evidentiary quality of these SRs/MAs has not been assessed, and it remains controversial whether these findings could provide credible evidentiary support for clinical staff [15]. The SR/MA overview is a newly emerged approach that combines multiple SRs/MAs to assess their quality and various findings in an attempt to resolve inconsistencies between them [16]. The purpose of this overview is to objectively and comprehensively evaluate the scientific validity and applicability of SRs/MAs regarding the effects of TC exercise on T2DM.

## 2. Methods

### 2.1. Research Methods

The SR/MA overview is based on the guidelines specified in the *Cochrane Handbook* [17], and we followed the methods of Shi et al. [18], Liu et al. [19], and Shen et al. [20].

### 2.2. Eligibility Criteria

#### 2.2.1. Literature Inclusion Criteria

*(1) Type of Research*. This overview includes SRs/MAs of randomized controlled trials (RCTs) of the effects of TC exercise on T2DM.

*(2) Type of Participants*. Subjects were patients diagnosed with T2DM by any international or national standard.

*(3) Type of Intervention*. The intervention for the control group was usual care or standard treatment (ST) or any type of other exercises, and the intervention for the experimental group was TC exercise or TC combined with the treatments received by the control group.

*(4) Types of Outcomes*. Outcomes assessed in this overview include fasting blood glucose (FBG), glycated hemoglobin (HbA1c), homeostasis model assessment of insulin resistance (HOMA-IR), fasting serum insulin (FINs), postprandial blood glucose (PBG), low-density lipoprotein (LDL), triglycerides (TG), high-density lipoprotein (HDL), total cholesterol (TCL), diastolic blood pressure (DBP), systolic blood pressure (SBP), body mass index (BMI), and quality of life.

#### 2.2.2. Exclusion Criteria

The exclusion criteria were as follows: (1) animal studies and (2) network MAs, research protocols, narrative reviews, overviews, dissertation, and conference abstracts.

### 2.3. Data Sources and Search Strategy

The literatures were retrieved from PubMed, Cochrane Library, Embase, Chongqing VIP, Wanfang Database, CNKI, and SINOMED on January 1, 2022. We adopted a strategy that combines keyword search with free word search, and the keywords include “Type 2 Diabetes Mellitus”, “Tai Chi”, “Systematic Review”, and “Meta-Analysis”. The literature search strategy of the PubMed database is shown in Table 1, which was reasonably tuned for each database. We also reviewed the references of all retrieved literatures to avoid missing topic-related SRs/MAs.

### 2.4. Literature Screening and Data Extraction

The literature screening (HS-S and YF-Z) and information extraction (PL-L and CD-C) were performed independently by two researchers. We imported the retrieved documents into Endnote X9 document management software and removed the duplicates. The literatures that potentially met the inclusion and exclusion criteria were then obtained by reading the titles and abstracts of these literatures. Ultimately, we finalized the included SRs/MAs by reading the full text. All SRs/MAs were read by two independent researchers, and the following data were extracted from the SRs/MAs: first author, publication year, country, number of RCTs included, interventions for experimental and control groups, included RCT quality assessment tools, and main conclusion. The disagreement between the two researchers was resolved through discussion.

### 2.5. SR/MA Quality Assessment

Two researchers (HS-S and CD-C) independently assessed the methodological and evidentiary quality of the included SRs/MAs.

### 2.6. Assessment of Methodological Quality

#### 2.6.1. Estimate of Methodological Quality

The methodological quality of the included SRs/MAs was assessed by the Assessment System for Evaluating Methodological Quality 2 (AMSTAR-2) [21]. Seven (2, 4, 7, 9, 11, 13, and 15) of the 16 items in the tool were critical areas.

#### 2.6.2. Assessment of Risk of Bias

The Risk of Bias in Systematic Review (ROBIS) [22] scale was used in this overview to evaluate the risk of bias of the inclusion of SRs/MAs. The scale was used to assess the overall risk of bias in the inclusion of SRs/MAs in three stages.

#### 2.6.3. Assessment of Reporting Quality

The quality of each SR/MA report of the included SRs/MAs was evaluated by the list of PRISMA [23] which consisted of 27 items focusing on the reporting methods and results that were incorporated into the SRs/MAs.

#### 2.6.4. Assessment of Quality of Evidence

The quality of evidence for each SR/MA outcome was evaluated by the Grading of Recommendations Assessment, Development, and Evaluation (GRADE) [24], according to which, five aspects will lead to the degradation of evidence quality, including limitations, inconsistencies, indirectness, imprecision, and publication bias.

## 3. Results

### 3.1. Results on Literature Search and Selection

**Figure 1:**
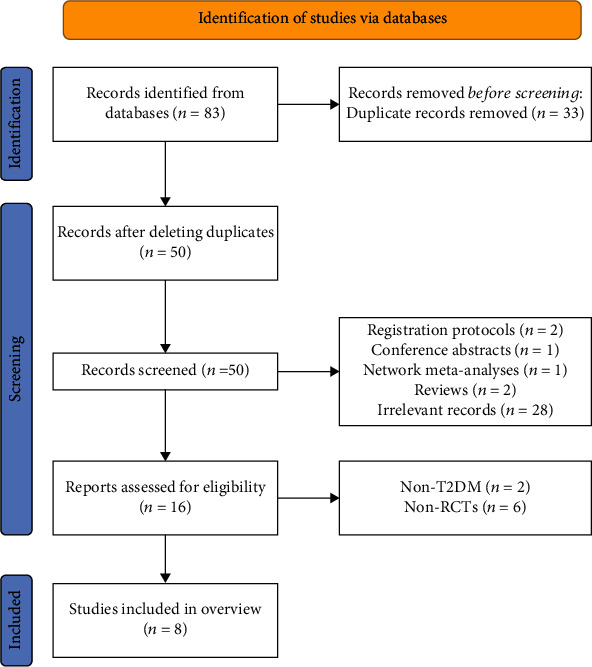
The flowchart of the screening process.

Using our search strategy, a total of 83 articles were identified. After removing 33 duplicate articles, the researchers screened the remaining 50 articles by reading titles and abstracts. Subsequently, 16 articles were obtained. After reading the full text, 6 articles [25–30] were found irrelevant to SRs/MAs in RCTs, and 2 SRs/MAs [31, 32] were not about people with T2DM. Thus, 8 SRs/MAs [33–40] were finally included in this overview. The process of study selection is shown in Figure 1.

### 3.2. Description of Included SRs/MAs

The characteristics included in the overview are shown in Table 2. These SRs/MAs were all published between 2017 and 2021, 5 [33–37] of which were in English, and the remaining 3 [38–40] were in Chinese. One [34] of the SRs/MAs was published by Korean researchers, and the remaining 7 SRs/MAs [33, 35–40] were published by Chinese researchers. The number of RCTs was between 10 and 23, and the sample size was between 740 and 1,800. In terms of quality evaluation scales, 6 SRs/MAs [34–36, 38–40] used the Cochrane risk of bias standard, 1 SR/MA [37] used the Physiotherapy Evidence Database scale, and 1 SR/MA [33] used the Jadad scale.

### 3.3. Results on SR/MA Quality Assessment

#### 3.3.1. Methodological Quality Assessment

Regarding the methodological quality of the included SRs/MAs, all were considered to be of very low quality because more than one key item was missing from the SRs/MAs included in the quality assessment. Methodological quality limitations come from the following items: item 2 (none of the SR/MA registers the study protocol), item 7 (no SR/MA provided by SR/MA provided for exclusion), and item 13 (when interpreting the evaluation results, only 2 SRs/MAs [34, 36] considered the risk of bias in the main study). The evaluation details of the included SRs/MAs on the AMSTAR-2 are shown in Table 3.

#### 3.3.2. Risk of Bias of the Included SRs/MAs

Regarding the results of the ROBIS assessment, phase 1 assessed the relevance of the study topic and domain 1, and all SRs/MAs were rated as low risk of bias in both items. Five [33–37] of the SRs/MAs were assessed as low risk in domain 2, 5 SRs/MAs [33, 35–37, 39] were assessed as low risk of bias in domain 3, and only 3 SRs/MAs [33, 36, 39] were assessed as low risk of bias in domain 4. In phase 3, only 2 SRs/MAs [34, 36] had a low risk of bias. The evaluation details of the included SRs/MAs on the ROBIS scale are shown in Table 4.

#### 3.3.3. Report Quality of the Included SRs/MAs

The results of the PRISMA inventory evaluation are shown in Table 5. 22 out of 27 items have a “yes” response rate of more than 70%, and this shows that the report was relatively complete. However, there were some reporting deficiencies in other items. The reports of item 5 (protocol and registration), item 8 (search), and item 15 (risk of bias across studies, methods) were incomplete (the “yes” response rate was less than 50%).

#### 3.3.4. Evidence Quality of the Included SRs/MAs

The 8 SRs/MAs included 65 outcomes related to the effectiveness of TC for T2DM. By means of the GRADE evaluation, 1 was rated as high quality, 7 moderate quality, 10 low quality, and 47 very low quality in terms of the quality of evidence for all outcome indicators. Risk of bias (*n* = 58) was the most common downgrading factor, followed by publication bias (*n* = 47), inconsistency (*n* = 37), imprecision (*n* = 37), and indirectness (*n* = 0). GRADE-specific assessment details are shown in Table 6.

### 3.4. Summary Results of the Included Studies

The result indicators extracted from the included studies are listed in Table 6.

#### 3.4.1. Outcomes Related to Glucose Metabolism

Eight SRs/MAs [33–40] reported the effect of TC on FGB in patients with T2DM, and all SRs/MAs indicated that TC could significantly reduce FGB. However, 3 subreports [33–35] also showed that compared with other aerobic exercises, TC had no significant effect on FGB. Eight SRs/MAs [33–40] reported the effect of TC on HbA1c in T2DM patients, of which 7 SRs/MAs [33, 35–40] indicated that TC could significantly reduce HbA1c levels. Two SRs/MAs [33, 35] reported the effect of TC on PBG in T2DM patients. Two reports [33, 35] showed that TC could significantly reduce PBG level, and 1 report [33] showed that TC had no significant effect on PBG compared with other aerobic exercises. Two SRs/MAs [33, 37] reported the effect of TC on FINs, and one SR/MA [35] showed that TC was beneficial for lowering FINs. In addition, 2 SRs/MAs [33, 37] reported that TC reduced HOMA-IR in T2DM patients.

#### 3.4.2. Outcome-Related Lipid Metabolism

Six SRs/MAs [35–40] reported the effect of TC on TCL in T2DM patients, of which 4 reports [35–37, 40] showed that TC could significantly reduce TCL level, while 3 reports [35, 38, 39] showed that the effect of TC on TCL was not statistically significant. Five SRs/MAs [35, 36, 38–40] reported the effect of TC on TG in T2DM patients. Five reports [35, 36, 38–40] suggested that TC could significantly reduce TG level, while 1 report [35] showed that TC had no significant effect on TG compared with other aerobic exercises. Four SRs/MAs [35, 36, 38, 39] reported the effect of TC on LDL in patients with T2DM, of which 2 SRs/MAs [36, 38] showed that TC could significantly lower LDL levels. Four SRs/MAs [35, 36, 38, 39] reported the effect of TC on HDL in patients with T2DM, and 3 of the SRs/MAs [35, 38, 39] indicated that TC could significantly improve HDL levels.

#### 3.4.3. Other Outcomes

Four SRs/MAs [35–37, 40] reported that TC reduced BMI in T2DM patients. One SR/MA [37] reported that TC reduced SBP and DBP in T2DM patients. Two SRs/MAs [37, 40] reported that TC could improve the quality of life of patients with T2DM. One SR/MA [37] result showed that TC had no statistically significant effect on balance.

#### 3.4.4. Adverse Event

There are 3 SR/MA narrative reviews suggesting that TC is safe.

## 4. Discussion

Exercise therapy is recommended for T2DM management because regular physical activity improves glycemic control as well as lipid index, blood pressure, cardiovascular disease, and quality of life [41]. TC is a low-to-moderate-intensity mind-body exercise that originated in China and is very popular around the world [42]. This overview is the first comprehensive and systematic assessment of the SRs/MAs associated with TC intervention for T2DM and will help to establish a clear link between the need to resolve uncertainty and prior clinical knowledge [43].

### 4.1. Summary of the Main Findings

This overview includes 8 SRs/MAs on the impact of TC on T2DM. All SRs/MAs are based on RCTs and published from 2015 to 2021. Among them, 7 (7/8, 87.5%) SRs/MAs were published in the past five years, indicating that the improvement effect of TC on T2DM has attracted increasing attention over the years.

Based on the results of the AMSTAR-2 evaluation in this overview, the methodological quality of all SRs/MAs was assessed as very low, especially in item 2 (protocol registration, 0/8, 0%), item 7 (exclusion list, 0/8, 0%), and item 13 (RoB account, 2/8, 25%). None of the SR/MA registered study protocols. No SR/MA contained initial research protocol registrations, which could lead to greater than expected adjustments to the research process, increasing the risk of bias and impacting the rigor and credibility of the final SR/MA results [44]. None of the SR/MA provides a complete exclusion of the lists for each study, which may affect the reliability of the results and assessment of publication bias. Providing a list of exclusion researches is a more strong demonstration of the rigor of the literature screening process. The authors of the 6 SRs/MAs did not consider the risk of bias of including RCTs when interpreting or discussing the study results, which may reduce the reliability of the final results. The risk of bias assessment of the ROBIS scale indicated that only one SR/MA was at low risk. Further analysis revealed that inadequate interpretation of the risk of bias and inadequate evaluation of publication bias were the main factors contributing to the high risk of bias. Similar to the results of the AMSTAR-2, the PRISMA assessment results indicate a lack of registration of programs. In addition, the included literatures only provided search keywords without elaborating specific search strategies, which reduces the reproducibility and credibility of the research.

According to the evidence quality assessment for the 65 outcomes by means of GRADE, 1 was rated as high quality, 7 moderate quality, 10 low quality, and 47 very low quality. Further generalization revealed the following common pitfalls in the inclusion of RCTs: only randomization was mentioned, without elaborating specific randomization method; allocation was not concealed; and only single blinding was performed. Therefore, the low methodological quality of the included trials was the underlying factor contributing to the decline of evidence quality. Besides, the lack of publication bias assessment was also an important reason for the downgrading of the evidence quality. The high heterogeneity of relevant outcome measures may be related to the unreasonable design of the original study. In addition, the insufficient scale included in the RCTs was also an important downgrading factor. Descriptive analysis showed that TC is an effective and safe method for the treatment of T2DM. Due to the low quality of methodology and evidence from the included studies, the conclusions of SRs/MAs may differ from real results, so we cannot draw firm conclusions about TC for T2DM.

### 4.2. Implications for Practice and Research

As a regular small-to-medium-intensity aerobic exercise, the main benefit of TC is not to consume calories but to promote the metabolism of cells and tissues, enhance cardiopulmonary function, activate antioxidant and anti-inflammatory activities, promote blood return to the heart, and improve the body's ability to respond to glucose. It can improve the utilization rate of target cells, improve the body's glucose tolerance, prevent the composition of HbA1c, and accelerate the combination of hemoglobin and oxygen [45]. In addition to this, the antioxidant effect of TC can be explained by the excitatory process, in which TC acts as a moderate exercise and the continuous stimulation of ROS generated by the adaptive process promotes the antioxidant response [46]. In this case, the main associated proteins are MAPK and FNkB [47] and the antioxidant gene Nrf2. Regular exercise leads to the upregulation of endogenous antioxidant defenses and counteracts the harmful effects of ROS [48]. Given the nature of TC exercise, the antioxidant effects may be attributed not only to the described excitatory processes but also to the interplay among several mechanisms related to meditation and diaphragmatic respiration. Studies have shown that psychological stress is positively correlated with increased production of free radicals and OS [49]. As for diaphragmatic breathing exercise, one study showed that the relaxation induced by diaphragmatic breathing increases the antioxidant defense status of athletes after exhaustive exercise [50].

In conclusion, when using the AMSTAR-2, PRISMA, ROBIS, and GRADE assessments to assess and standardize various aspects of the included SR/MA, researchers are expected to register or publish research protocols in advance when conducting SRs/MAs to minimize the risk of bias and ensure the accuracy of SRs/MAs results, and they should also provide a list of excluded literatures to ensure transparency and avoid publication bias. For literatures at high risk of bias, researchers should conduct separate analyses and provide reasonable explanations to ensure the quality of the evidence. In addition, a complete assessment of publication bias would also improve the accuracy of the meta-analysis results. Although the peculiarities of TC therapy can make blinding difficult to implement, a carefully designed and rigorously implemented RCT can minimize or avoid bias, which is the gold standard for evaluating interventions [51]. Clinical researchers should improve the top-level design of clinical trials through comprehensive evaluation and sophisticated analysis. Notably, Consolidated Standards of Reporting Trials (CONSORT) should be used to improve the quality of evidence from RCTs [52]. In view of the low evidence quality regarding the effect of TC exercise on T2DM, researchers should strictly describe all stages of their research, specify the research scheme, and register study protocols in their future researches, so as to facilitate publication and subsequent inclusion in SRs/MAs. At the same time, during SRs/MAs, researchers should also strictly follow relevant methodologies to improve the evidence system.

Although TC originated from traditional Chinese medicine theory, the duration, frequency, and mode of TC movement vary greatly in different studies. Therefore, we propose to use a standardized TC training program, where duration, frequency, and pattern are formalized, so as to better study the impact of TC on T2DM. Researchers should also pay attention to the effect of TC on related biochemical indicators in T2DM patients, including indicators related to OS. In addition to this, most studies focused on the therapeutic effects of TC on T2DM, but it still remains unclear whether TC can reduce the risk of associated complications in individuals with T2DM. Further research on the relationship between TC and the risk of developing various complications of T2DM is recommended.

### 4.3. Strength and Limitations

Our overview is the first to use AMSTAR2, ROBIS, PRISMA, and GRADE to evaluate SRs/MAs regarding the impact of TC on T2DM. Based on the current results, TC may be an effective adjunctive replacement therapy for T2DM. Furthermore, the evaluation process revealed clear limitations of the current relevant SRs/MAs and RCTs, which may help guide future high-quality clinical studies. However, this overview has certain limitations because the assessment is subjective. While our assessments were assessed and reviewed by two independent assessors, different assessors may have their personal judgment on each factor, so the results may vary.

## 5. Conclusion

In conclusion, TC is beneficial and safe for T2DM. However, due to the generally low methodological and evidentiary quality of the included SRs/MAs, clinicians should approach these findings with caution in their practice.

## Figures and Tables

**Table 1:** Search strategy for the PubMed database.

Query	Search term
#1	“Tai Ji”[Mesh]
#2	“Tai-ji” OR “Tai Chi” OR “Chi, Tai” OR “Tai Ji Quan” OR “Ji Quan, Tai” OR “Quan, Tai Ji” OR “Taiji" OR “Taijiquan” OR “T'ai Chi” OR “Tai Chi Chuan” OR “Tai ji”
#3	#1 OR #2
#4	“Diabetes Mellitus, Type 2”[Mesh]
#5	“Diabetes Mellitus, Noninsulin-Dependent” OR “Diabetes Mellitus, Ketosis-Resistant” OR “Diabetes Mellitus, Ketosis Resistant” OR “Ketosis-Resistant Diabetes Mellitus” OR “Diabetes Mellitus, Non Insulin Dependent” OR “Diabetes Mellitus, Non-Insulin-Dependent” OR “Non-Insulin-Dependent Diabetes Mellitus” OR “Diabetes Mellitus, Stable” OR “Stable Diabetes Mellitus” OR “Diabetes Mellitus, Type II” OR "NIDDM” OR “Diabetes Mellitus, Noninsulin Dependent” OR “Diabetes Mellitus, Maturity-Onset” OR “Diabetes Mellitus, Maturity Onset” OR “Maturity-Onset Diabetes Mellitus” OR “Maturity Onset Diabetes Mellitus” OR “MODY” OR “Diabetes Mellitus, Slow-Onset” OR “Diabetes Mellitus, Slow Onset” OR “Slow-Onset Diabetes Mellitus” OR “Type 2 Diabetes Mellitus” OR “Noninsulin-Dependent Diabetes Mellitus” OR “Noninsulin Dependent Diabetes Mellitus" OR “Maturity-Onset Diabetes” OR “Diabetes, Maturity-Onset” OR “Maturity Onset Diabetes" OR “Type 2 Diabetes” OR “Diabetes, Type 2” OR “Diabetes Mellitus, Adult-Onset" OR “Adult-Onset Diabetes Mellitus” OR “Diabetes Mellitus, Adult Onset”
#6	#4 OR #5
#7	Meta-Analysis as Topic [Mesh]
#8	“Systematic review” OR “meta-analysis” OR “meta analysis” OR “meta-analyses” OR “Review, Systematic”
#9	#7 OR #8
#10	#3 AND #6 AND #9

**Table 2:** Characteristics of the included SRs/MAs. Note: N: nonexercise; AE: aerobic exercise; ST: standard treatment.

Author, year (country)	Trials (subjects)	Intervention group	Control group	Quality assessment	Main results
Mengyao Chao, 2018 (China) [33]	14 (798)	TC	N, AE	Jadad	TC can effectively influence the management of blood glucose and HbA1c in patients with T2DM. Long-term adherence to TC has a better effect on reducing blood sugar and HbA1c levels in patients with T2DM
Myeong Soo Lee, 2015 (South Korea) [34]	15 (754)	TC, TC+control group	N, ST, AE	Cochrane criteria	In conclusion, the evidence that TC may benefit people with T2DM compared with exercise therapy is not convincing. In addition, evidence from RCTs comparing TC with conventional antidiabetic drugs appears to be mixed
Shuai Guo, 2021 (China) [35]	23 (1,800)	TC, TC+control group	ST, AE	Cochrane criteria	Compared with routine clinical treatment, TC has better effects on blood sugar control, lipid metabolism, and body composition and is superior to aerobic exercise in improving partial metabolic control. The optimal intervention time window for TC may vary for different metabolic markers
Ting-Wei Xia, 2019 (China) [36]	17 (774)	TC, TC+control group	N, ST, AE	Cochrane criteria	TC appears to be effective in treating T2DM compared to control interventions. Different training times and methods will lead to differences in effects
Zonglei Zhou, 2019 (China) [37]	23 (1,235)	TC	AE, ST	PEDro scale	TC was effective in controlling biomedical outcomes and improving the quality of life-related outcomes in patients with T2DM, but no effects on balance and fasting insulin were observed
Yao Ge, 2020 (China) [38]	13 (856)	TC, TC+control group	N, ST	Cochrane criteria	TC exercise can control blood sugar level and regulate lipoprotein concentration in patients with T2DM, which can provide the basis for exercise therapy for later stage diabetes
Yongjin Liu, 2017 (China) [39]	10 (740)	TC	N, AE	Cochrane criteria	TC exercise can regulate the level of glucose and lipid metabolism and improve the quality of life in patients with T2DM and can be used as an important part of exercise therapy for diabetes
Qing Tang, 2017 (China) [40]	11 (764)	TC	CT	Cochrane criteria	TC helps improve blood sugar control, weight loss, blood lipid regulation, and quality of life in patients with T2DM

**Table 3:** Result of the AMSTAR-2 assessments. Note: Y: yes; PY: partial yes; N: no; VL: very low; L: low. Note: key items are marked in italic.

Author, year (country)	Q1	*Q2*	Q3	*Q4*	Q5	Q6	*Q7*	Q8	*Q9*	Q10	*Q11*	Q12	*Q13*	Q14	*Q15*	Q16	Quality
Mengyao Chao, 2018 (China) [33]	Y	PY	Y	Y	Y	Y	N	Y	Y	N	Y	Y	N	Y	Y	Y	VL
Myeong Soo Lee, 2015 (South Korea) [34]	Y	PY	Y	Y	Y	Y	N	Y	Y	N	Y	Y	Y	Y	N	Y	VL
Shuai Guo, 2021 (China) [35]	Y	PY	Y	Y	Y	Y	N	Y	Y	N	Y	Y	N	Y	N	Y	VL
Ting-Wei Xia, 2019 (China) [36]	Y	PY	Y	Y	Y	Y	N	Y	Y	N	Y	Y	Y	Y	Y	Y	VL
Zonglei Zhou, 2019 (China) [37]	Y	PY	Y	Y	Y	Y	N	Y	Y	N	Y	Y	N	Y	Y	Y	VL
Yao Ge, 2020 (China) [38]	Y	PY	Y	PY	Y	Y	N	N	Y	N	Y	Y	N	Y	N	Y	VL
Yongjin Liu, 2017 (China) [39]	Y	PY	Y	PY	Y	Y	N	Y	Y	N	Y	Y	N	Y	Y	Y	VL
Qing Tang, 2017 (China) [40]	Y	PY	Y	PY	Y	Y	N	Y	Y	N	Y	Y	N	Y	N	Y	VL

**Table 4:** Results of the ROBIS assessments. Note: √: low risk; ×: high risk.

Author, year (country)	Phase 1	Phase 2				Phase 3
	Assessing relevance	Domain 1: study eligibility criteria	Domain 2: identification and selection of studies	Domain 3: collection and study appraisal	Domain 4: synthesis and findings	Risk of bias in the review
Mengyao Chao, 2018 (China) [33]	√	√	√	√	√	×
Myeong Soo Lee, 2015 (South Korea) [34]	√	√	√	×	×	√
Shuai Guo, 2021 (China) [35]	√	√	√	√	×	×
Ting-Wei Xia, 2019 (China) [36]	√	√	√	√	√	√
Zonglei Zhou, 2019 (China) [37]	√	√	√	√	×	×
Yao Ge, 2020 (China) [38]	√	√	×	×	×	×
Yongjin Liu, 2017 (China) [39]	√	√	×	√	√	×
Qing Tang, 2017 (China) [40]	√	√	×	×	×	×

**Table 5:** Results of the PRISMA checklist. Note: Y: yes; N: no.

Section/topic	Items	Mengyao Chao, 2018 (China) [33]	Myeong Soo Lee, 2015 (South Korea) [34]	Shuai Guo, 2021 (China) [35]	Ting-Wei Xia, 2019 (China) [36]	Zonglei Zhou, 2019 (China) [37]	Yao Ge, 2020 (China) [38]	Yongjin Liu, 2017 (China) [39]	Qing Tang, 2017 (China) [40]	Number of yes (%)
Title	Q1. Title	Y	Y	Y	Y	Y	Y	Y	Y	100%

Abstract	Q2. Structured summary	Y	Y	Y	Y	Y	Y	Y	Y	100%

Introduction	Q3. Rationale	Y	Y	Y	Y	Y	Y	Y	Y	100%
	Q4. Objectives	Y	Y	Y	Y	Y	Y	Y	Y	100%

Methods	Q5. Protocol and registration	N	N	N	N	N	N	N	N	0%
	Q6. Eligibility criteria	Y	Y	Y	Y	Y	Y	Y	Y	100%
	Q7. Information sources	Y	Y	Y	Y	Y	Y	Y	Y	100%
	Q8. Search	N	N	Y	N	Y	N	N	N	25%
	Q9. Study selection	Y	Y	Y	Y	Y	Y	Y	Y	100%
	Q10. Data collection process	Y	Y	Y	Y	Y	Y	Y	Y	100%
	Q11. Data items	Y	Y	Y	Y	Y	Y	Y	Y	100%
	Q12. Risk of bias in individual studies	Y	Y	Y	Y	Y	Y	Y	Y	100%
	Q13. Summary measures	Y	Y	Y	Y	Y	Y	Y	Y	100%
	Q14. Synthesis of results	Y	Y	Y	Y	Y	Y	Y	Y	100%
	Q15. Risk of bias across studies	N	N	N	Y	Y	N	N	N	25.00%
	Q16. Additional analyses	Y	N	Y	Y	Y	N	N	Y	62.50%

Results	Q17. Study selection	Y	Y	Y	Y	Y	Y	Y	Y	100%
	Q18. Study characteristics	Y	Y	Y	Y	Y	N	Y	Y	88%
	Q19. Risk of bias within studies	Y	Y	Y	Y	Y	Y	Y	Y	100%
	Q20. Results of individual studies	Y	Y	Y	Y	Y	Y	Y	Y	100%
	Q21. Synthesis of results	Y	Y	Y	Y	Y	Y	Y	Y	100%
	Q22. Risk of bias across studies	Y	N	N	Y	Y	N	Y	N	50.00%
	Q23. Additional analysis	Y	N	Y	Y	Y	N	Y	Y	75.00%

Discussion	Q24. Summary of evidence	Y	Y	Y	Y	Y	Y	Y	Y	100%
	Q25. Limitations	Y	Y	Y	Y	Y	Y	Y	Y	100%
	Q26. Conclusions	Y	Y	Y	Y	Y	Y	Y	Y	100%

Funding	Q27. Funding	Y	Y	Y	Y	Y	Y	Y	Y	100%

**Table 6:** Results of evidence quality. ^①^The included studies have a large bias in methodology such as randomization, allocation concealment, and blinding. ^②^The confidence interval overlaps less, or the *I*^2^ value of the combined results was larger. ^③^The sample size from the included studies does not meet the optimal sample size, or the 95% confidence interval crosses the invalid line. ^④^The funnel chart is asymmetry. ^∗^The 95% confidence interval does not cross the invalid line.

Author, year (Country)	Outcomes	Studies (participants)	Limitations	Inconsistency	Indirectness	Imprecision	Publication bias	Relative effect (95% CI)	Quality
Mengyao Chao, 2018 (China) [28]	FBG (tai chi versus nonexercise)	10 (489)	-1^①^	-1^②^	0	0	-1^④^	MD = −1.39 (-1.95, -0.84)^∗^	Very low
	FBG (tai chi versus other aerobic exercises)	7 (342)	-1^①^	-1^②^	0	-1^③^	-1^④^	MD = −0.21 (-0.61, 0.19)	Very low
	HbA1c (tai chi versus nonexercise)	7 (293)	-1^①^	0	0	-1^③^	-1^④^	MD = −0.73 (-0.95, -0.52)^∗^	Very low
	HbA1c (tai chi versus other aerobic exercises)	7 (372)	-1^①^	-1^②^	0	-1^③^	-1^④^	MD = −0.19 (-0.37, 0.00)^∗^	Very low
	PBG (tai chi versus nonexercise)	5 (162)	-1^①^	0	0	-1^③^	-1^④^	MD = −2.07 (-2.89, -1.26)^∗^	Very low
	PBG (tai chi versus other aerobic exercises)	3 (84)	-1^①^	0	0	-1^③^	-1^④^	MD = −0.44 (-1.42, 0.54)	Very low

Myeong Soo Lee, 2015 (South Korea) [29]	HbA1c (tai chi versus ST)	3 (127)	-1^①^	0	0	-1^③^	-1^④^	MD = −0.54 (−1.23, 0.15)	Very low
	HbA1c (tai chi versus other aerobic exercises)	2 (148)	-1^①^	0	0	-1^③^	-1^④^	MD = 0.00 (−0.31, 0.31)	Very low
	HbA1c (no)	2 (84)	-1^①^	-1^②^	0	-1^③^	-1^④^	MD = −1.58 (−3.83, 0.67)	Very low
	FBG (tai chi versus ST)	4 (188)	-1^①^	0	0	-1^③^	-1^④^	MD = −1.57 (−2.34, −0.80)^∗^	Very low
	FBG (tai chi versus other aerobic exercises)	4 (212)	-1^①^	0	0	-1^③^	-1^④^	MD = −0.03 (−0.49, 0.42)	Very low

Shuai Guo, 2021 (China) [30]	FBG (tai chi versus ST)	15 (1,023)	-1^①^	-1^②^	0	0	-1^④^	MD = −1.04 (-1.42, -0.66)^∗^	Very low
	FBG (tai chi versus other aerobic exercises)	8 (619)	-1^①^	-1^②^	0	-1^③^	-1^④^	MD = −0.03 (-0.30, 0.23)	Very low
	HbA1c (tai chi versus ST)	9 (749)	-1^①^	-1^②^	0	0	-1^④^	MD = −0.73 (-1.03, -0.43)^∗^	Very low
	HbA1c (tai chi versus other aerobic exercises)	5 (504)	-1^①^	0	0	0	-1^④^	MD = −0.33 (-0.61, 0.04)^∗^	Low
	PBG (tai chi versus ST)	2 (260)	-1^①^	0	0	-1^③^	-1^④^	MD = −1.58 (-1.94,-1.22)^∗^	Very low
	TCL (tai chi versus ST)	11 (868)	-1^①^	-1^②^	0	0	-1^④^	MD = −0.51 (-0.88, -0.14)^∗^	Very low
	TCL (tai chi versus other aerobic exercises)	5 (423)	-1^①^	0	0	-1^③^	-1^④^	MD = −0.08 (-0.24, 0.09)	Very low
	TG (tai chi versus ST)	9 (745)	-1^①^	-1^②^	0	0	-1^④^	MD = −0.40 (-0.72, -0.07)^∗^	Very low
	TG (tai chi versus other aerobic exercises)	4 (332)	-1^①^	-1^②^	0	-1^③^	-1^④^	MD = 0.04 (−0.22, 0.31)	Very low
	HDL (tai chi versus ST)	9 (798)	-1^①^	-1^②^	0	0	-1^④^	MD = 0.39 (0.14, 0.63)^∗^	Very low
	HDL (tai chi versus other aerobic exercises)	5 (538)	-1^①^	0	0	0	-1^④^	MD = 0.24 (0.07, 0.41)^∗^	Low
	LDL (tai chi versus ST/other aerobic exercises)	9 (730)	-1^①^	-1^②^	0	0	-1^④^	MD = −0.79 (-1.27, -0.30)^∗^	Very low
	BMI (tai chi versus ST)	5 (358)	-1^①^	0	0	0	-1^④^	MD = −1.15 (-1.79, -0.51)^∗^	Low
	SBP (tai chi versus ST)	5 (390)	-1^①^	0	0	-1^③^	-1^④^	MD = −11.86 (-14.47, -9.25)^∗^	Very low
	DBP (tai chi versus ST)	5 (390)	-1^①^	-1^②^	0	-1^③^	-1^④^	MD = −7.93 (-12.39, -3.46)^∗^	Very low
	FINs (tai chi versus ST)	3 (255)	-1^①^	0	0	-1^③^	-1^④^	MD = −1.02 (-1.39, -0.64)^∗^	Very low
	HOMA-IR (tai chi versus ST)	3 (255)	-1^①^	-1^②^	0	-1^③^	-1^④^	MD = −0.65 (-1.01, -0.30)^∗^	Very low

Ting-Wei Xia, 2019 (China) [31]	FBG	13 (616)	-1^①^	-1^②^	0	0	-1^④^	SMD = −0.54 (-0.91, -0.16)^∗^	Very low
	HbA1c	9 (517)	-1^①^	-1^②^	0	0	-1^④^	SMD = −0.68 (-1.17, -0.19)^∗^	Very low
	TCL	8 (343)	-1^①^	0	0	-1^③^	-1^④^	SMD = −0.35 (-0.54, -0.16)^∗^	Very low
	TG	8 (359)	-1^①^	0	0	-1^③^	-1^④^	SMD = −0.19 (-0.31, -0.07)^∗^	Very low
	HDL	6 (290)	-1^①^	0	0	-1^③^	-1^④^	SMD = 0.04 (− 0.01, 0.09)	Very low
	LDL	6 (290)	-1^①^	-1^②^	0	-1^③^	-1^④^	SMD = −0.49 (− 1.06, 0.08)	Very low
	BMI	6 (296)	-1^①^	0	0	-1^③^	-1^④^	SMD = −0.61 (− 0.85, − 0.38)^∗^	Very low

Zonglei Zhou, 2019 (China) [32]	FBG	21 (1,115)	-1^①^	-1^②^	0	0	0	SMD = −0.67 (-0.87, -0.47)^∗^	Low
	HbA1c	12 (714)	0	0	0	0	0	MD = −0.53 (-0.62, -0.44)^∗^	High
	FINs	8 (500)	-1^①^	-1^②^	0	-1^③^	0	SMD = −0.32 (-0.71, 0.07)	Very low
	HOMA-IR	5 (332)	0	0	0	-1^③^	0	MD = −0.41 (-0.78, -0.04)^∗^	Moderate
	TCL	10 (658)	-1^①^	-1^②^	0	0	0	SMD = −0.59 (-0.90, -0.27)^∗^	Low
	BMI	7 (388)	0	0	0	-1^③^	0	MD = −0.82 (-1.28, -0.37)^∗^	Moderate
	Balance	2 (107)	0	-1^②^	0	-1^③^	0	MD = 2.71 (-3.29, 8.71)	Low
	SBP	5 (290)	0	-1^②^	0	-1^③^	0	MD = −10.03 (-15.78, -4.29)^∗^	Low
	DBP	5 (290)	0	0	0	-1^③^	0	MD = −4.85 (-8.23, -1.47)^∗^	Moderate
	Physical function	5 (389)	-1^①^	-1^②^	0	-1^③^	0	MD = 7.07 (0.79, 13.35)^∗^	Very low
	Bodily pain	5 (389)	-1^①^	0	0	-1^③^	0	MD = 4.30 (0.83, 7.77)^∗^	Low
	Social function	6 (426)	0	-1^②^	0	0	0	MD = 13.84 (6.22, 21.47)^∗^	Moderate

Yao Ge, 2020 (China) [33]	FBG	9 (560)	-1^①^	-1^②^	0	0	-1^④^	SMD = −0.607 (-0.930, -0.284)^∗^	Very low
	HbA1c	7 (434)	-1^①^	-1^②^	0	0	-1^④^	SMD = −0.585 (-0.784, -0.386)^∗^	Very low
	TCL	7 (533)	-1^①^	-1^②^	0	-1^③^	-1^④^	SMD = −0.418 (-0.897, 0. 061)	Very low
	TG	6 (480)	-1^①^	-1^②^	0	0	-1^④^	SMD = −0.833 (-1.383, -0.283)^∗^	Very low
	HDL	4 (420)	-1^①^	-1^②^	0	0	-1^④^	SMD = 0.458 (0. 063, 0.852)^∗^	Very low
	LDL	3 (76)	-1^①^	-1^②^	0	-1^③^	-1^④^	SMD = −1.252 (-2.305, -0.199)	Very low

Yongjin Liu, 2017 (China) [34]	FBG	9 (727)	-1^①^	0	0	0	0	SMD = −0.39 (-0.54, -0.24)^∗^	Moderate
	HbA1c	7 (645)	-1^①^	0	0	0	0	MD = −0.59 (-0.73, -0.44)^∗^	Moderate
	TCL	7 (612)	-1^①^	-1^②^	0	-1^③^	0	SMD = −0.24 (-0.58, 0.10)	Very low
	TG	7 (612)	-1^①^	-1^②^	0	0	0	SMD = −0.52 (-0.85, -0.19)^∗^	Low
	HDL	6 (566)	-1^①^	0	0	0	0	SMD = 0.31 (0.14, 0.47)^∗^	Moderate
	LDL	6 (462)	-1^①^	-1^②^	0	0	0	MD = −0.32 (-0.59, -0.05)^∗^	Low

Qing Tang, 2017 (China) [35]	FBG	7 (354)	-1^①^	-1^②^	0	-1^③^	-1^④^	MD = −0.74 (-1.32, -0.16)^∗^	Very low
	HbA1c	7 (572)	-1^①^	-1^②^	0	0	-1^④^	MD = −0.77 (-1.16, -0.39)^∗^	Very low
	BMI	4 (316)	-1^①^	0	0	-1^③^	-1^④^	MD = −1.64, (-2.35, -0.92)^∗^	Very low
	TG	6 (518)	-1^①^	-1^②^	0	0	-1^④^	MD = −0.33 (-0.49, -0.17)^∗^	Very low
	TCL	6 (518)	-1^①^	-1^②^	0	0	-1^④^	MD = −0.08 (-0.33, -0.48)^∗^	Very low
	Quality of Life	2 (264)	-1^①^	0	0	-1^③^	-1^④^	MD = 45.47 (18.24, 72.71)^∗^	Very low

## Data Availability

The datasets analyzed during the current study are available from the corresponding author on reasonable request.
